# A Double-Taper Optical Fiber-Based Radiation Wave Other than Evanescent Wave in All-Fiber Immunofluorescence Biosensor for Quantitative Detection of *Escherichia coli* O157:H7

**DOI:** 10.1371/journal.pone.0095429

**Published:** 2014-05-07

**Authors:** Zhonghuan Zhang, Fei Hua, Ting Liu, Yong Zhao, Jun Li, Ruifu Yang, Changxi Yang, Lei Zhou

**Affiliations:** 1 State Key Laboratory of Precision Measurement Technology and Instrument, Department of Precision Instruments, Tsinghua University, Beijing, P.R. of China; 2 Laboratory of Analytical Microbiology, State Key Laboratory of Pathogen and Biosecurity, Beijing Institute of Microbiology and Epidemiology, Beijing, P.R. of China; 3 Department of Aetiology, Taishan Medical University, Taian, P.R. of China; Semmelweis University, Hungary

## Abstract

Cylindrical or taper-and-cylinder combination optical fiber probe based on evanescent wave has been widely used for immunofluorescence biosensor to detect various analytes. In this study, in contrast to the contradiction between penetration depth and analyte diameter of optical fiber probe-based evanescent wave, we demonstrate that double-taper optical fiber used in a radiation wave-based all-fiber immunofluorescence biosensor (RWAIB) can detect micron-scale analytes using *Escherichia coli* O157:H7 as representative target. Finite-difference time-domain method was used to compare the properties of evanescent wave and radiation wave (RW). Ray-tracing model was formulated to optimize the taper geometry of the probe. Based on a commercial multi-mode fiber, a double-taper probe was fabricated and connected with biosensor through a “ferrule connector” optical fiber connector. The RWAIB configuration was accomplished using commercial multi-mode fibers and fiber-based devices according to the “all-fiber” method. The standard sample tests revealed that the sensitivity of the proposed technique for *E*. *coli* O157:H7 detection was 10^3^ cfu·mL^−1^. Quantitation could be achieved within the concentration range of 10^3^ cfu·mL^−1^ to 10^7^ cfu·mL^−1^. No non-specific recognition to ten kinds of food-borne pathogens was observed. The results demonstrated that based on the double-taper optical fiber RWAIB can be used for the quantitative detection of micron-scale targets, and RW sensing is an alternative for traditional evanescent wave sensing during the fabrication of fiber-optic biosensors.

## Introduction

Since the application of fiber optics in sensing technology in the late 1970s [1]–[2], optical fiber has been established as an ideal substrate for immunofluorescence sensing because of their reliability, small size, and low cost [3]. Immunofluorescence sensing has great potential for rapid and sensitive analysis of various analytes, ranging from molecules to intact cells, and as an effective technique for biodefense, environmental monitoring, food-security control, etc. [4]–[5]. Most fiber optic biosensors utilize the evanescent wave (EW) field generated by the total internal reflection (TIR) of light restrained within an optical fiber as sensing region. In this sensing region, specifically bound fluorescence-labeled antibodies can be excited to emit light collected as specific signal, whereas the unbound ones outside the sensing region cannot be excited, thereby adding nothing to the noise. Given this natural separation, fiber-optic biosensors have high signal-to-noise ratio [6], and they are used for the detection of various small analytes [7] and microorganisms [8]–[9].

The development of fiber-optic biosensors has recently received much attention. In hardware design, bulk optical components [10]–[11], such as biconvex lens and dichroic mirrors are common bases in implementing this type of biosensors. Analyte 2000 [8], [12] and RAPTOR [13], two commercial instruments developed using miniaturized and integrated bulk optical components, showed great potential in immunofluorescence sensing, but their optical alignment and precise processing technology remain challenging. Given the rapid development of highly integrated optical components, an “all-fiber” method is used to fabricate more compact fiber-optic biosensors [7] and enables crucial optical alignment no longer necessary. In terms of mechanism of fluorescence sensing, most fiber-optic biosensors utilize EW to generate the excitation field regardless of the continuous development of hardware components. Taper-and-cylinder combination tapered probes are usually used to generate EW field [7], [13] and prepared using a tube-etching technique [14]. To enhance the sensitivity of EW excitation, U-bent optical fiber probes were used [15]–[16]. However, the fiber probe with definite structure has fixed incident ray angle to stimulate EW and refractive indices of core and cladding and then the characteristics of generated EW cannot be tuned as that of waveguide-based EW [17]–[20], especially penetration depth (PD). PD provides the inherent advantage of natural separation to EW for fluorescence sensing with high signal-to-noise ratio, but limits its superiority when the diameter of the detected target is larger than the PD [6]. Based on the numerical aperture (NA) of fibers, PD of all-fiber biosensors is usually several hundreds of nanometers [21], which hardly exceeds the size of bacteria (1 µm to 5 µm diameter, or larger). Therefore, fluorescein-labeled antibodies bound on bacteria surface but beyond the PD of EW field cannot be detected, resulting in an inevitable loss of sensitivity. Although several simulations have demonstrated that the PD value could be several micrometers in a specially designed fiber probe, a large PD value only exists within ≤5% of the entire probe length, leaving a major portion invalid [22]. This condition might result in poor consistency of quantitation at low concentrations of the detected target. Additionally, EW that penetrates from the probes only accounts for a small part of the entire excitation light power, which leads to a low percentage of utilization of the excitation light [23].

In the present study, finite-difference time-domain (FDTD) method was used to compare the properties of EW and radiation wave (RW). A ray-tracing model was formulated to optimize the taper geometry of the optical fiber probe. Based on a commercial multi-mode fiber, a double-taper optical fiber probe was proposed and fabricated as a low-cost, disposable sensing unit for RW sensing. The sensing unit was connected to the biosensor through a “ferrule connector (FC)” optical fiber connector. Therefore, the consistency and stability of these sensing units for practical tests could be guaranteed by the reliable telecommunication fiber connection technology. Subsequently, a novel RW-based all-fiber immunofluorescence biosensor (RWAIB) was developed to analyze simultaneously the specific signals within and beyond the reach of EW. The configuration of the RWAIB was developed using commercial multi-mode fibers and fiber-based devices according to the “all-fiber” method. The comprehensive performance including quantitation ability, sensitivity, and specificity of RWAIB was estimated using *Escherichia coli* O157:H7 as the representative micron-scale target/analyte.

## Materials and Methods

### Reagents

3-Aminopropyl-triethoxysilane (APTES), glutaraldehyde, and bovine serum albumin (BSA) were purchased from Sigma-Aldrich (Gillingham, Dorset, UK). Cyanine 5 (Cy5) and HiTrap Desalting prepacked column were obtained from GE Healthcare (Uppsala, Sweden). Unless otherwise specified, all reagents, which were provided by Sinopharm Chemical Reagent Co., Ltd. (Shanghai, China), were of analytical grade and used without further purification. Deionized water was used throughout the experiment.

### Bacterial Culture and Antibody Preparation

*E. coli* O157:H7, *Salmonella choleraesuis, Salmonella enteritidis, Salmonella paratyphi* A, *S. paratyphi* B, *S. paratyphi* C, *Salmonella typhi, Salmonella typhimurium, Vibrio parahaemolyticus, Vibrio cholerae* O1, and *V. cholerae* O139 were previously preserved in our laboratory and identified using 16sRNA sequencing. The bacteria were grown until they reached the exponential phase in Luria-Bertani (LB) media at 37°C. The bacteria were harvested by centrifugation at 6000 rpm (Allegra X-22R, Beckman, Germany) for 10 min at 4°C. The bacterial pellets were washed twice and resuspended with sterile normal saline (0.85% salt solution). Bacterial concentration was determined using plate count and demonstrated as colony forming units (cfu) per milliliter.

A monoclonal antibody (MAb) specific for *E. coli* O157:H7 was prepared in our laboratory, and its affinity was determined using *E. coli* O157:H7-coated ELISA. MAb was labeled with Cy5 and purified with HiTrap Desalting prepacked column to separate and clear free Cy5 and antibody molecules, according to the manufacturer’s instruction.

### Double-taper Probe Fabrication, Modification and Activation

The double-taper probe designed to generate RW was made from a length of step-index optical fibers (105 µm core/125 µm cladding, Beijing Glass Research Institute, Beijing, China). At the non-sensing end, an FC standard optical fiber connector was fixed to facilitate the alignment of the sensor optical path during the repeated installation-and-uninstallation of the probes. At the sensing end, a double-tapered structure was fabricated using a simple static-and-dynamic etching method with 40% HF as the etchant (experimental details are provided as File S1); this method combined classical static tube etching [24] and dynamic liquid-level-lowering etching [25] and promoted the large-scale preparation of probes with good uniformity. The fabricated double-taper probe was bathed successively in NaOH (1 mol·L^−1^) and HCl (1 mol·L^−1^) for 10 min, and then dried for future use. The calibrated optical microscopic images show that the diameter of taper 1 of fabricated double-taper probe was etched from 125 µm to ∼40 µm within the length of ∼270 µm with the *V* number matching the diameter [21]; the diameter of taper 2 was reduced to 26 µm at distal end within the length of ∼2.5 cm.

The double-taper probe was silanized by immersing it in 10% APTES (in isopropyl alcohol) for 2 h. As a result, a monolayer silane film was covalently bonded on the silica surface of the probe with the amino functional groups on top. Subsequently, the silanized probe was functionalized using an amine-reactive homo-bifunctional cross-linker glutaraldehyde (12.5%, in deionized water) for 2 h. Residual glutaraldehyde was rinsed with phosphate-buffered saline (PBS; 135 mmol·L^−1^ NaCl, 15 mmol·L^−1^ sodium phosphate, pH 7.2). The aldehyde group-activated probe was then incubated in MAb solution (0.5 mg·mL^−1^ in PBS) for 4 h at 37°C. Finally, the probe was immersed in BSA solution (1 mg·mL^−1^ in PBS) for 30 min to block non-specific binding cites. The activated sensing probes were stored at 4°C for future use.

### RWAIB Structural Design

**Figure 1:**
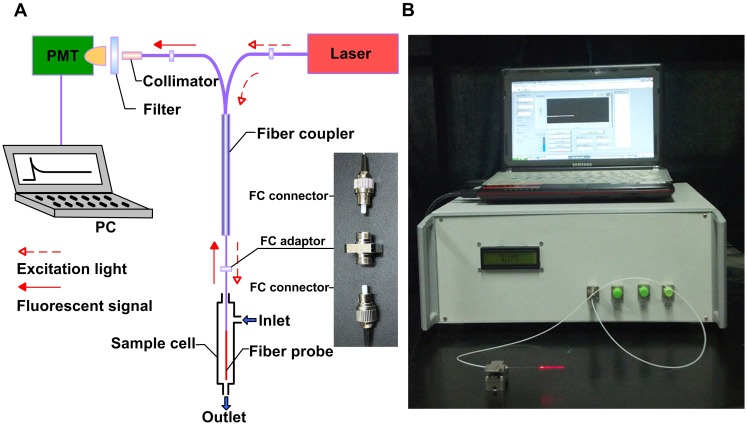
Schematic diagram (A) and prototype (B) of all-fiber biosensor. (A) Twenty percent of the 643 nm excitation light was conducted from the semiconductor laser to the fiber probe through a fiber coupler with FC connectors as the linker. Eighty percent of 668 nm fluorescent signal was collected and transmitted back from the fiber probe to the PMT through a fiber coupler, fiber collimator, and high-pass filter. The signal was processed and displayed on the computer screen. (B) The prototype of all-fiber biosensor consisted of an integrated detecting unit and a controlling unit (computer). RW field produced the visible red light around the fiber probe.

A semiconductor laser with wavelength of 643 nm and tunable output power of 20 mW (Shanghai Fiblaser Technology Co., Ltd, Shanghai, China) was selected as excitation light source, because the excitation wavelength of Cy5 is at 646 nm (Figure 1). The pigtail of the laser was linked to one input end of an optical fiber coupler (1×2, couple ratio: 20/80, Beijing Glass Research Institute, Beijing, China). Twenty percent of the input power could be transmitted to the fiber probe through the coupler. The fiber probe was connected to the coupler using a “FC” optical fiber connector (inset in Figure 1A), which is a common device in optical fiber telecommunication with insertion and return loss that are almost the same in different individuals. The 643 nm light formed an RW field around the fiber probe to excite Cy5 that was bonded on the surface. The fluorescent signal with 668 nm emission wavelength was collected by the fiber probe, and 80% of signal power was sent back to the other input end of the coupler. Fluorescent signal was filtered using a high-pass filter and injected into a photomultiplier tube (PMT-CR131, Beijing Hamamatsu Photon Techniques Inc., Beijing, China) through a fiber collimator. The signal was processed by an electronic system and then displayed on a computer screen. During the entire process of detection, the probe was installed in a 4-mm diameter, 50-mm long poly-propylene sample cell.

### Evaluation of RWAIB Performance in Detecting *E. coli* O157:H7

To determine the sensitivity (detection limit) and quantitation ability of the technique, water samples containing various concentrations (10^3^ cfu·mL^−1^ to 10^7^ cfu·mL^−1^) of *E. coli* O157:H7 were used as standard solutions, with each sample detected in triplicate. The samples were separately injected into the sample cell from its inlet and incubated for 10 min at room temperature to achieve the specific capture of bacteria by the antibody-activated probe. After the transfer of the sample from the outlet of the sample cell, the cleaning buffer (0.1% Tween20 in PBS) was injected into the cell, and the nonspecific binding and residual sample in the cell were removed. Subsequently, Cy5-labelled antibodies (25 µg·mL^−1^) were injected into the sample cell and incubated for 10 min at room temperature, then transferred out through the tubule. Immediately after another cycle of the cleaning process, the laser was turned on and maintained for 150 s while signal data was monitored, recorded, and displayed. After biosensor analysis, the surface state of probe with adhering bacteria was observed under a scanning electron microscope (S-3400 N, Hitachi, Japan).

Ten kinds of food-borne pathogens, namely, *S. enteritidis*, *S. paratyphi* A, *S. paratyphi* B, *S. paratyphi* C, *S. typhimurium, S. choleraesuis, S. typhi, V. parahaemolyticus, V. cholerae* O1, and *V. cholerae* O139, were tested using the biosensor at 10^7^ cfu·mL^−1^ to estimate the specificity of the technique.

## Results and Discussion

### EW and RW Sensing Mechanism

Light transmission within optical fiber is based on the principle of TIR. Light beams propagating inside the fiber core with incident angles (*α*) greater than the critical incident angle (*α_c_*) can be guided along the fiber, where *α_c_* is determined using the refractive indices of the fiber core and cladding [*α_c_* = sin^−1^(*n_cl_/n_co_*)]. When TIR occurs, EW exists beyond the reflecting interface, and the electric field intensity of EW decays exponentially with the distance from the interface. The PD of EW generally refers to the distance at which the magnitude of electric field at the surface decays to its 1/*e* value [26]. However, if the light beams hit the interface with incident angles less than *α_c_*, they will be partially propagated inside the core and partially transmitted into the surrounding medium. In partial transmission, the beams experience reflection and transmission simultaneously and disappear after a short propagating distance. This short-distance propagating wave is called RW [27]. Unlike EW, RW is not restricted near the surface but emitted into surroundings, so that the extended sensing region and added excitation power provide the potential to increase the accuracy and sensitivity of the quantitative detection, especially for analytes with diameter beyond 1 µm.

### Simulation of EW and RW

Given that incident angles (*α*) of different beams are constant in a cylindrical probe because of the law of reflection, RW cannot exist for long distances. However, tapered probe is suitable for generating RW because it can gradually decrease incident angles while the beams propagate along it. Thus, all beams can generate RW.

**Figure 2:**
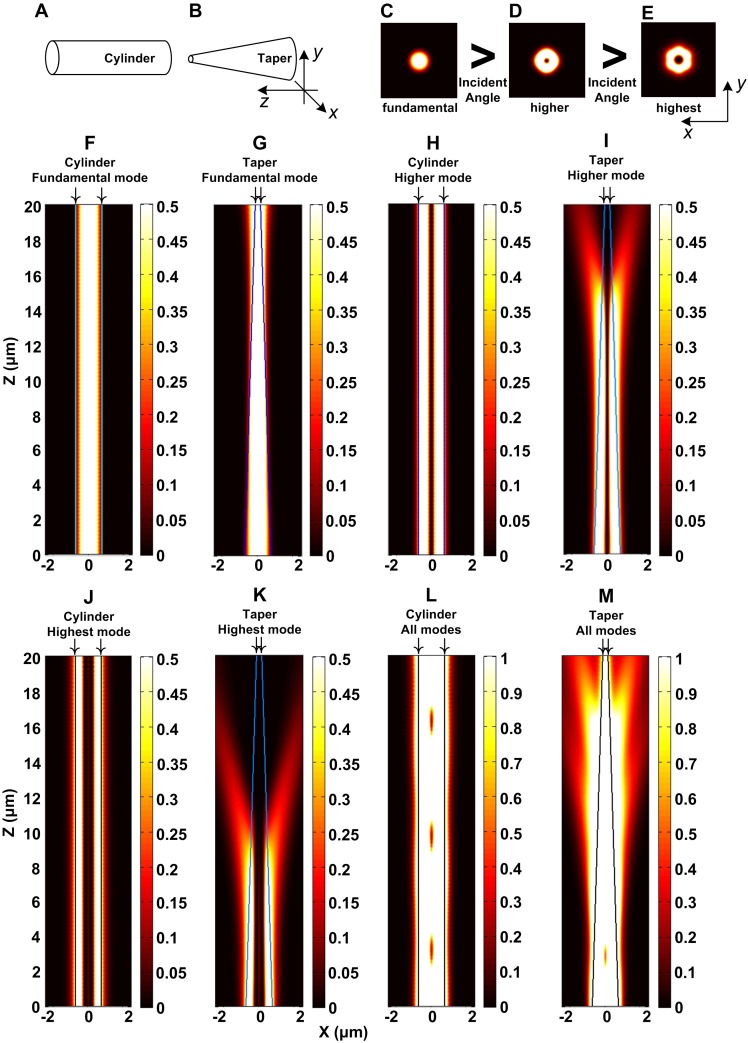
FDTD calculation results of the EW and RW generated by the cylindrical and tapered probes. (A) and (B) are simplified models of the cylindrical and tapered probes; (C), (D), and (E) are the three guided modes of light in the cylindrical probe (A) in increasing order; (F), (H), and (J) are the axial cross-sections (*x*–*z* planes) of the cylindrical probe as the light under modes (C), (D), and (E) propagates in the probe separately; (G), (I), and (K) are the axial cross-sections (*x*–*z* planes) of the tapered probe as the light under modes (C), (D), and (E) propagates in the probe separately; (L) and (M) are the axial cross-sections (*x*–*z* planes) of the cylindrical and tapered probes as the light under all of the modes propagates in the probes simultaneously. Downward arrows (↓) indicate the outlines of the probes.

Simplified models of EW and RW generated by cylindrical (Figure 2A) and tapered probes (Figure 2B), respectively, were calculated using FDTD method. The radius of the cylindrical probe was 0.75 µm, whereas that of the tapered probe was reduced from 0.75 µm to 0.15 µm, and both models had lengths of 20 µm. The refractive indices of the probes (silica) and surrounding medium (water) were 1.456 and 1.333, respectively. The three guided modes of light in the cylindrical probe in successive order (i.e., the incident angles of these three modes decreased successively) are shown in Figures 2C, 2D, and 2E. The axial cross-sections (*x*–*z* planes) of the cylindrical probe as the light propagated in the probes under modes C, D, and E are shown in Figure 2F, 2H, and 2J, respectively. Downward arrows (↓) indicate the outlines of the probes, whereas the optical power penetrating outside the outlines is the EW field. PD increased as the mode order increased (incident angle decreased), but did not exceed hundreds of nanometers. The axial cross-sections (*x*–*z* planes) of the tapered probe as the light propagated in the probe under modes C, D, and E are shown in Fig. 2G, 2I, and 2K, respectively. The light under fundamental mode C was always restricted within the probe, but its PD increased as light propagated to the distal end. Light under higher mode D changed from guided to radiation mode and then generated RW after a certain distance of propagation. The light under the highest mode E generated RW before the light under mode D because of a smaller incident angle. The axial cross-sections (*x*–*z* planes) of the cylindrical and tapered probes when the light propagates under all of the modes simultaneously are shown in Figs. 2L and 2M, which demonstrated that the RW could offer a much larger sensing (or excitation) region than the EW. Additionally, the tapered probe, transforming guided mode with various order to radiation mode, enabled the RW distribution throughout the entire length of the probe instead of a short distance (Figures 2M). Therefore, a tapered probe is suitable for generating RW in detecting micron-scale analytes, which could not be thoroughly covered by the EW sensing region.

### Ray-tracing in the Tapered Probe

**Figure 3:**
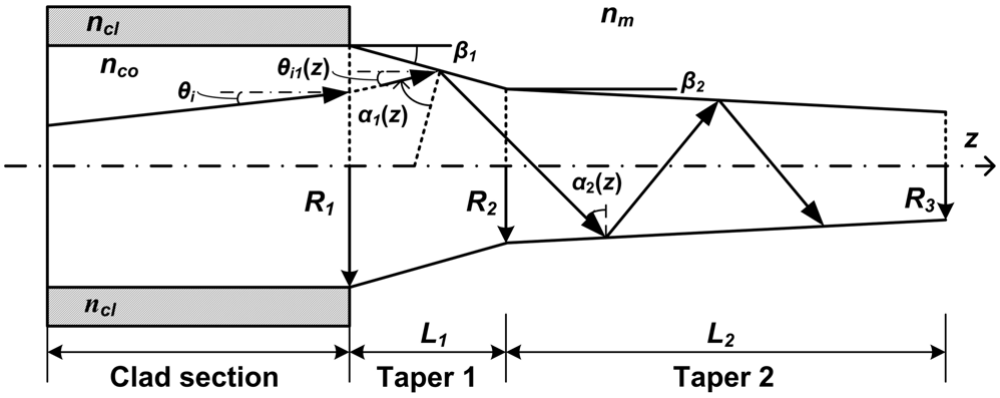
Ray tracing model of a tapered probe.

The incident angle (*α*) is the critical factor that determines the generation of RW of a tapered probe. When *α*≤*α_c_*, the guided wave changes into RW; otherwise, only the EW penetrates outside the probe. To characterize the incident angles *α*(*z*) in the tapered fiber probe, a ray-tracing model was established (Figure 3).

The core radius of a step index fiber with core and cladding refractive indices of *n_co_* and *n_cl_*, respectively, was etched from *R_1_* to *R_2_* to form taper 1, and then successively etched from *R_2_* to *R_3_* to form taper 2. The lengths of these two tapers were *L_1_* and *L_2_*, and their half cone angles were *β_1_* and *β_2_*, respectively. The refractive index of the medium surrounding the tapered probe was *n_m_*.

For continuous tapered probe (i.e., taper 2 is not considered), a guided ray from clad section at an angle of *θ_i_* with respect to the *z*-axis was launched into taper 1, and reflected at position *z*, where the launch angle is changed into *θ_i1_*(*z*). According to reference [28], *θ_i1_*(*z*) = sin^−1^[*R_1_*sin*θ_i_*/*R*(*z*)], where *R*(*z*) = *R_1_*–*z* tan*β_1_*. Hence, the incident angle in taper 1, *α_1_*(*z*), can be given as.
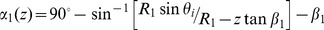
(1)As *α_1_*(*z*) decreased into less than *α_c_* after a TIR propagating distance of *L_1_*_(*α*≥*αc*)_, RW started to appear where the core radius was reduced to *R_2_*_(*α* = *αc*)_. Given that NA = 0.22, *R*_1_ = 52.5 µm, *n_co_* = 1.456, *n_cl_* = 1.444, and *n_m_* = 1.333, the same as the actual parameters used in our sensor, *L_1_*_(*α*≥*αc*)_ for different *β_1_* values were calculated. Several results are listed in Table 1, in which *L_1_*_(*tot*)_ is the complete length of taper 1 at a certain *β_1_* if taper 2 does not exist.

**Table 1:** *L_1_*_(*α*≥*αc*)_, *R_2_*_(*α*  =  *αc*)_, and *L_1_*_(*tot*)_ at different *β_1_*values.

*β_1_* a (°)	*L_1_*_(*a≥ac*)_ b (µm)	*R_2_*_(*a = ac*)_ c (µm)	*L_1_*_(*tot*)_ d (µm)
0.05	37591	19.70	60761
0.1	18773	19.73	30080
0.5	3717	20.06	6016
1	1834	20.49	3008
5	318	24.68	600
10	109	33.28	298

a Half cone angles of taper 1.

b TIR propagating distance alone taper 1 when *α* is equal to or greater than *α_c_*.

c Core radius of taper 1 where *α* is equal to *α_c_*.

d Complete length of taper 1.

Table 1 shows that the continuous tapered probe is not suitable for RW sensing because the invalid length [*L_1_*_(*α*≥*αc*)_] consists of more than 60% of the total length ranging from 3 mm (3008 µm) to 6 cm (60761 µm). The taper-and-cylinder combination tapered probe is also unsuitable for RW sensing because *α_2_*(*z*) is constant in the cylindrical section, and RW disappears at the start of this section after a short-distance propagation. Therefore, double-taper probe is necessary for RW sensing. Taper 1 is used to reduce *α_1_*(*z*) that is smaller than *α_c_* in a short length to avoid a long invalid length, whereas taper 2 is used to release RW power gradually and continually reduces the *α*_2_(*z*) of the other beams with lower mode order to generate new RW. The double-taper probe used in this paper can generate RW almost from the beginning of taper 2 to the distal end.

### Detection Data Analysis

**Figure 4:**
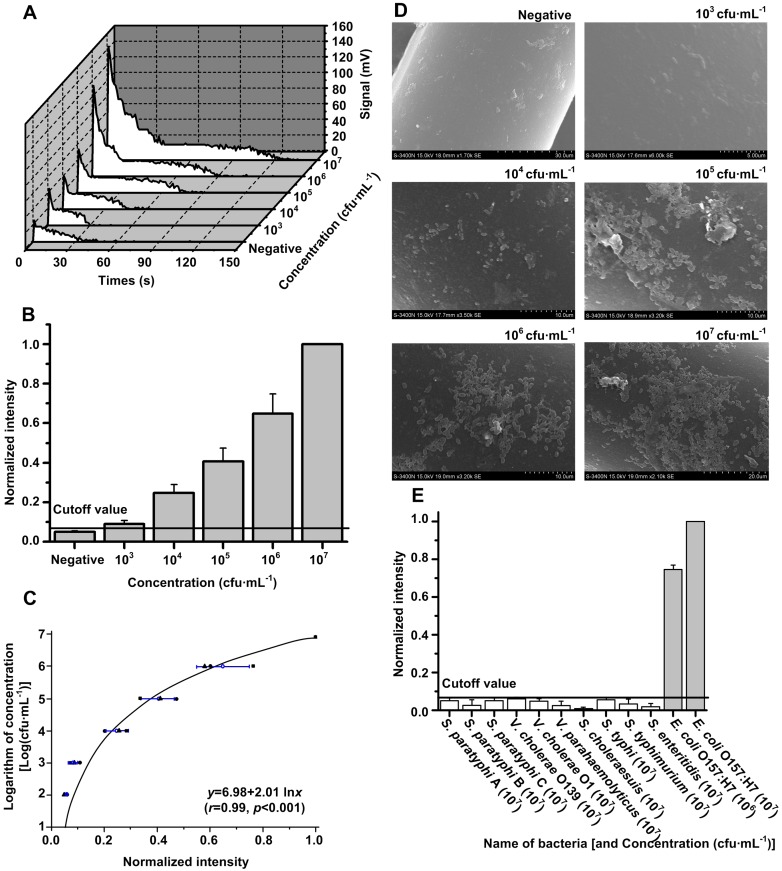
Evaluation of RWAIB for the quantitative detection of *E. coli* O157:H7. (A) A group of typical signal-time traces at different concentrations of *E. coli* O157:H7 during a complete test cycle (150 s). (B) Sensitivity for *E. coli* O157:H7 detection, where *x* is the concentration of bacteria, and *y* is the NI. The bar graph revealed a significant difference between positive sample (10^3^ cfu·mL^−1^ to 10^7^ cfu·mL^−1^) and negative control with the cutoff value determined as mean+3 SD of the negative control NIs. The sensitivity was 10^3^ cfu·mL^−1^. (C) Quantitation ability for *E. coli* O157:H7 detection, where *x* is the NI, and *y* is the logarithm of concentration. The correlation and regression analyses revealed an exponent correlation between *x* and *y*, with *r* = 0.99 (*p*<0.05) in the quantitative range of 10^3^ cfu·mL^−1^ to 10^7^ cfu·mL^−1^. (D) The scanning electron microscope images of probes corresponding to negative and positive samples (with concentrations from 10^3^ cfu·mL^−1^ to 10^7^ cfu·mL^−1^) proved the direct proportion between the amount of adhering bacteria and the concentration of sample. (E) Specificity for *E. coli* O157:H7 detection. A significant difference was observed between the NIs of 10 different kinds of food-borne pathogens at 10^7^ cfu·mL^−1^ (white bars) and the NIs of *E. coli* O157:H7 samples at 10^6^ and 10^7^ cfu·mL^−1^ (gray bars) with cutoff value as the threshold.

A group of typical signal-time traces at different concentrations of detected target during a complete test cycle (150 s) are shown in Figure 4A. For each trace, signal intensity reached the maximum value immediately after the laser was turned on (0 s), and then gradually decreased back to the baseline because of fluorescence quenching. The integral intensity within the time of a complete test cycle (150 s) was regarded as an effective result for each test. Given that the batch-to-batch variation in the antibody-activated probe production is inevitable, a normalization of intensity was used to obtain comparable results of different tests. The normalized intensity (NI) is expressed as follows:
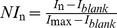
(2)where *I_n_* is the integral intensity for a certain test, *I*_max_ is the integral intensity for the maximum concentration of the detected target (10^7^ cfu·mL^−1^ for the detection of *E. coli* O157:H7), and *I*_blank_ is the integral intensity for the activated sensing probe in blank solution without any procedure for detection. For a certain batch of activated probe, *I*_max_ and *I*_blank_ were definitive.

### RWAIB Performance Evaluation for Detection of *E. coli* O157:H7

*E. coli* O157:H7 was selected as the representative micron-scale target to evaluate the performance of RWAIB, including the sensitivity (detection limit), quantitation ability (correlation analysis), and specificity of the technique. Water samples with 10^3^ cfu·mL^−1^ to 10^7^ cfu·mL^−1^ *E*. *coli* O157:H7, as well as a negative control (0 cfu·mL^−1^), were detected using RWAIB. NI against the concentration of the bacterium is shown in Figure 4B, with the integral intensity of 10^7^ cfu·mL^−1^ as *I*_max_. NIs of all positive samples (10^3^ cfu·mL^−1^ to 10^7^ cfu·mL^−1^) were significantly higher than the cutoff threshold (mean+3 SD of NIs corresponding to the negative control), which suggests that the sensitivity of RWAIB is 10^3^ cfu·mL^−1^. The regression curve for the quantitative detection of *E. coli* O157:H7 is shown in Figure 4C. An evident curvilinear correlation (exponent correlation) was found between *y* (logarithm of concentration) and *x* (NIs), in which the correlation coefficient (*r*) is equal to 0.99 (*p*<0.001) from 10^3^ cfu·mL^−1^ to 10^7^ cfu·mL^−1^. Regression analysis can be expressed as follows:
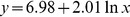
(3)


The scanning electron microscope images of the probes corresponding to negative and positive samples (with concentrations from 10^3^ cfu·mL^−1^ to 10^7^ cfu·mL^−1^) are shown in Figure 4D and the amount of the observed bacteria adhering on the surface of the probe is directly proportional to the increase in concentration. Ten kinds of food-borne pathogens at 10^7^ cfu·mL^−1^ were used to evaluate the specificity of RWAIB, using *E*. *coli* O157:H7 samples (10^6^ cfu·mL^−1^ and 10^7^ cfu·mL^−1^) as positive control. The high specificity of RWAIB, in which only two *E*. *coli* O157:H7 samples exhibited strong signals higher than the cutoff threshold, is illustrated in Figure 4E.

## Conclusion

Extensive studies have been conducted to develop and optimize static and dynamic chemical etching methods to control the taper length, cone angle, and geometry of the final optical fiber [29]–[33]. In these studies, the effects of fiber motion, etching rate, meniscus distortion, and etching time among others, have been explored. Geometrically optimized optical fibers, such as double-taper optical fiber, were used to ensure highly efficient light transmission for near-field scanning optical microscopy [29]–[30] and provide a surface-enhanced Raman scattering sensor with a large active surface and intensive internal reflections at the probe interface [31]–[33]. Based on the literature, our study first discussed the RW-producing property of double-taper optical fiber to resolve conflicts between PD and analyte diameter during EW-based fiber optic biosensing. We used FDTD calculation to intuitively demonstrate that the sensing region of RW is larger than that of EW.

The bio-active double-taper probes were fabricated from commercial communication multimode optical fibers using the traditional static-and-dynamic etching method, modified, and activated through a covalent method. Eventually, we have presented an all-fiber immunofluorescence biosensor with low-cost, disposable sensing units using double-taper probes for the quantitative detection of *E*. *coli* O157:H7. The standard sample tests revealed that the sensitivity of the technique for *E*. *coli* O157:H7 detection was 10^3^ cfu·mL^−1^, and quantitation could be achieved within the concentration range of 10^3^ cfu·mL^−1^ to 10^7^ cfu·mL^−1^. Non-specific recognition to other ten kinds of food-borne pathogens was not observed. Results demonstrate that the RWAIB can be used for the quantitative detection of micron-scale targets, and RW sensing is an alternative for traditional evanescent wave sensing during the fabrication of fiber-optic biosensors.

## Supporting Information

File S1 Double-taper probe fabrication. The static-and-dynamic etching device and its working process for double-taper probe fabrication are shown in details.
(DOC)
