# Supplementary material for: A Double-Taper Optical Fiber-Based Radiation Wave Other than Evanescent Wave in All-Fiber Immunofluorescence Biosensor for Quantitative Detection of Escherichia coli O157:H7
Source: PLoS One. 2014 May 7;9(5):e95429. doi: 10.1371/journal.pone.0095429 (PMC4013131; doi:10.1371/journal.pone.0095429)
Supplement: File S1 — Double-taper probe fabrication. The static-and-dynamic etching device and its working process for double-taper probe fabrication are shown in details. (DOC) [file pone.0095429.s001.doc]

**Double-taper probe fabrication**

The static-and-dynamic etching device and its working process are shown in Figure S1. Hydrofluoric acid (HF, 40%) was used as etchant, and the vessels, pipes, and valves made of Teflon were used for safety and stability. A potential energy difference existed between vessels 1 and 2 (vessel 1 > vessel 2). The two vessels were connected by a pipe with two valves. Valve 1 was used to control flow velocity of HF, whereas valve 2 was used as a flow switch. The tube-etching process was employed to construct taper 1 of the probe (Figure S1 A). Valve 2 was closed, and the two vessels both contained 40% HF with a liquid-level difference. A layer (~0.5 cm) of methylsilicone oil was laid over the surface of HF in both vessels to prevent volatilization. After striping the jackets and subsequent cleaning with alcohol, 4 cm length quartzose-fiber cores were vertically immersed into HF of vessel 1 for 25 min to form taper 1. The liquid-level-lowering etching process was proposed to build taper 2 (Figure S1 B). After valve 2 was opened, taper 2 was formed by the flow of HF from vessel 1 to vessel 2, and its half cone angle was regulated by the flow velocity that was controlled by valve 1. The fabricated double-taper probe was bathed successively in NaOH (1 mol·L^-1^) and HCl (1 mol·L^-1^) for 10 min, and then dried for future use. The calibrated optical microscopic images show that the diameter of taper 1 (Figure S1 C) was etched from 125 μm to ~40 μm within the length of ~270 μm with the *V* number matching the diameter. The diameter of taper 2 (Figure S1 D) was reduced to 26 μm at distal end within the length of ~2.5 cm.


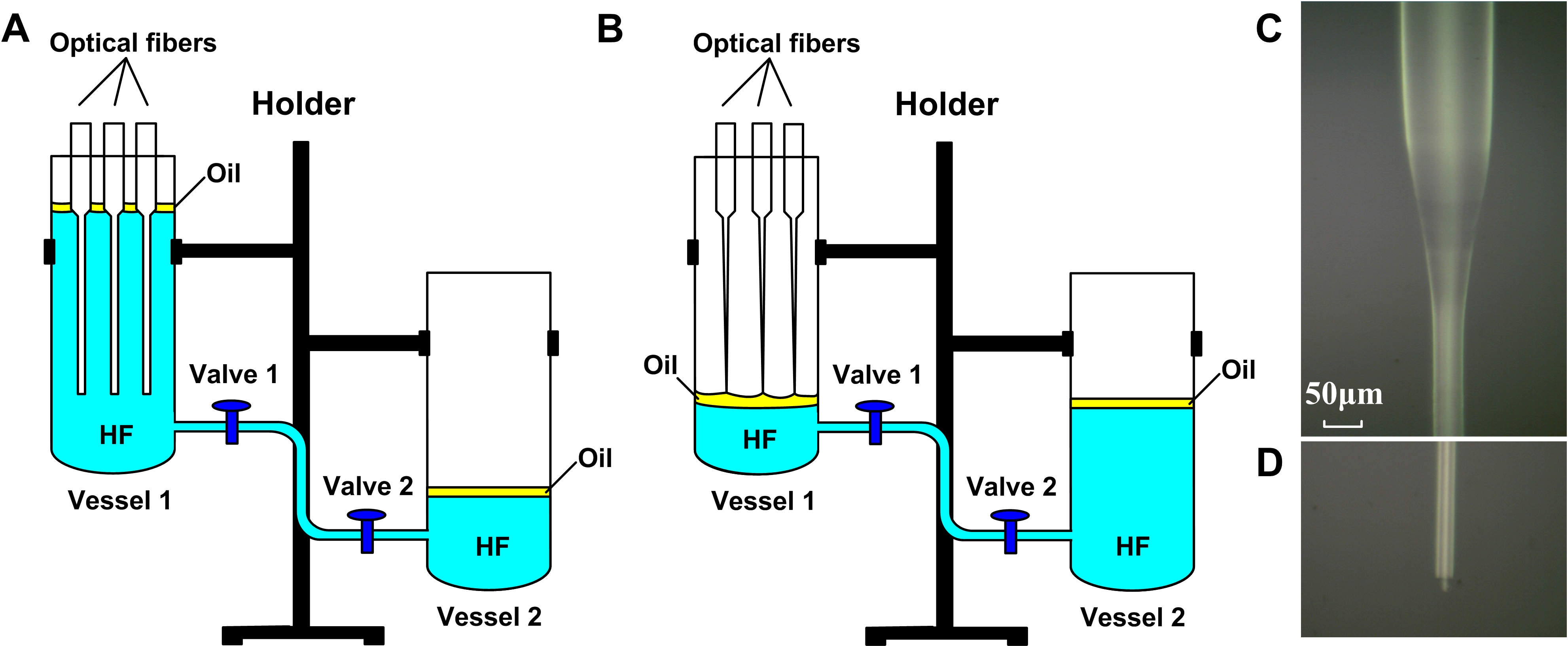


**Figure S1**

**Figure S1.** **The static-and-dynamic etching device and its working process for the fabrication of double-taper probe.** (A) When valve 2 was closed, the tube-etching (static) process with the static etchant was used to form taper 1. (B) When valve 2 was open and the flow velocity of etchant was appropriate under the control of valve 1, the liquid-level-lowering (dynamic) etching process with the flow of etchant from vessel 1 to vessel 2 was used to from taper 2. (C) The diameter of taper 1 was reduced from 125 μm to ~40 μm within the length of ~270 μm. (D) The diameter of taper 2 was reduced from ~40 μm to ~26 μm within the length of ~2.5 cm.
